# Fluorometric recognition of both dihydrogen phosphate and iodide by a new flexible anthracene linked benzimidazolium-based receptor

**DOI:** 10.3762/bjoc.7.34

**Published:** 2011-02-25

**Authors:** Kumaresh Ghosh, Debasis Kar

**Affiliations:** 1 Department of Chemistry, University of Kalyani, Kalyani-741235, India

**Keywords:** anthracene coupled receptor, benzimidazolium-based receptor, dihydrogenphosphate recognition, iodide recognition

## Abstract

A new flexible anthracene linked benzimidazolium-based receptor **1** has been designed, synthesized and its binding properties have been studied by NMR, UV–vis and fluorescence spectroscopic techniques. While the receptor **1** exhibits a greater change in emission in the presence of tetrabutylammonium dihydrogenphosphate in CH_3_CN over the other anions studied, iodide is selectively preferred in CHCl_3_ containing 0.1% CH_3_CN. Upon complexation of dihydrogen phosphate and iodide, the emission of **1** gradually decreased without showing any other characteristic change in the spectra. Hydrogen bonding and charge–charge interactions interplay simultaneously in a cooperative manner for selectivity in the binding process.

## Introduction

The selective recognition of anionic species by artificial abiotic receptors is a rapidly growing area in supramolecular chemistry [1–6]. Anion recognition has gained significant importance as it plays an important role in a wide range of biological, environmental and chemical processes [7–10]. Sensors based on anion-induced changes in fluorescence appear to be particularly attractive in anion recognition due to the simplicity and high detection limit of fluorescence [11–12]. In devising such sensors, various functional sites with hydrogen bond donors and acceptors are of considerable importance. Among the different binding motifs for anions, the hydrogen bonding properties of NH groups in neutral amines [13], amides [14], ureas/thioureas [15–16], indoles [17–19] and pyrroles [20] as well as in guanidinium [21] and imidazolium [22] groups are well established. In addition, the use of the benzimidazolium motif [23–24] in anion recognition is also known. During the course of our work on anion recognition, we used this motif along with the other functionalities for selective recognition of carboxylates, and dihydrogen phosphate [25]. Selective recognition of dihydrogen phosphate is an important aspect of supramolecular chemistry and various receptors of different designs for this anion have been reported in the literature [26–33].

**Figure 1:**
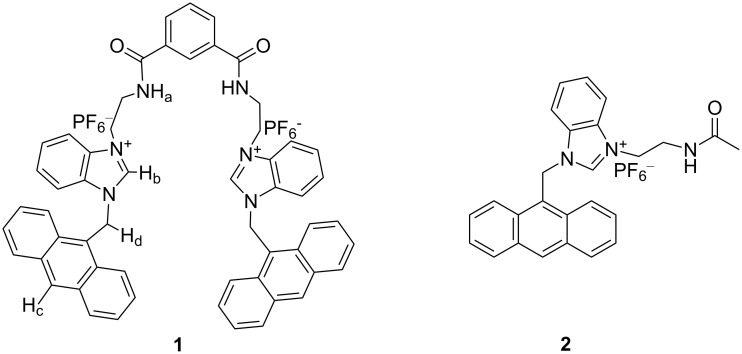
Synthesized compounds **1** and **2**.

In an effort to investigate the anion recognition behavior of the benzimidazolium group in the presence of a flexible spacer, we report herein the design and synthesis of a new chemosensor **1** (Figure 1), which shows selective recognition of dihydrogen phosphate in CH_3_CN among the other anions studied. The selectivity is changed on changing the polarity of the solvent, and the receptor **1** exhibits a preference for iodide when CH_3_CN is replaced by CHCl_3_ containing 0.1% CH_3_CN. The results were compared with the monomeric unit **2** (Figure 1).

## Results and Discussion

**Scheme 1:**
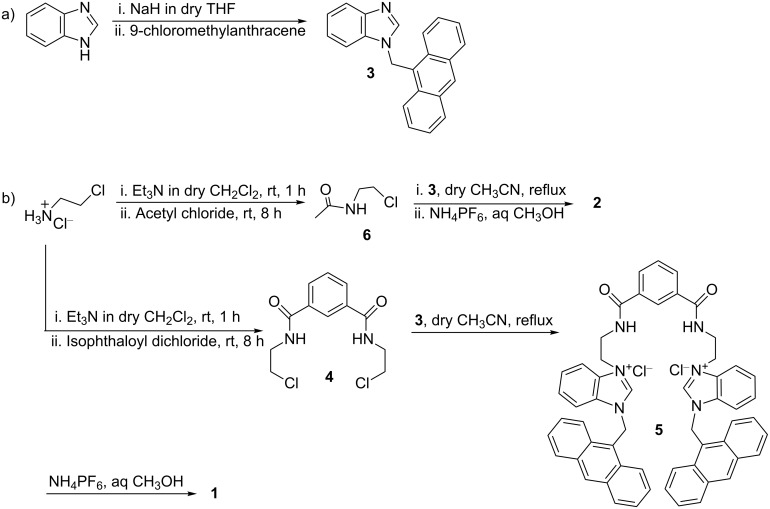
Synthesis of **1** and **2**.

The receptor **1** was obtained according to Scheme 1. Initially, 2-chloroethylamine was reacted with isophthaloyl dichloride to afford the diamide **4** in 80% yield. Subsequent reaction of **4** with anthracene coupled benzimidazole **3** (prepared in 64% yield from benzimidazole and 9-chloromethylanthracene in the presence of NaH in dry THF) gave the dichloride salt **5** in 55% yield. Anion exchange using NH_4_PF_6_ in warm aqueous CH_3_OH gave the desired compound **1** in 90% yield. Similarly, model compound **2** was obtained in 64% yield from the reaction between **6** and **3** in dry CH_3_CN followed by anion exchange using NH_4_PF_6_ in aqueous CH_3_OH. All the compounds were characterized unequivocally by ^1^H NMR, ^13^C, mass spectrometry and FTIR.

**Figure 2:**
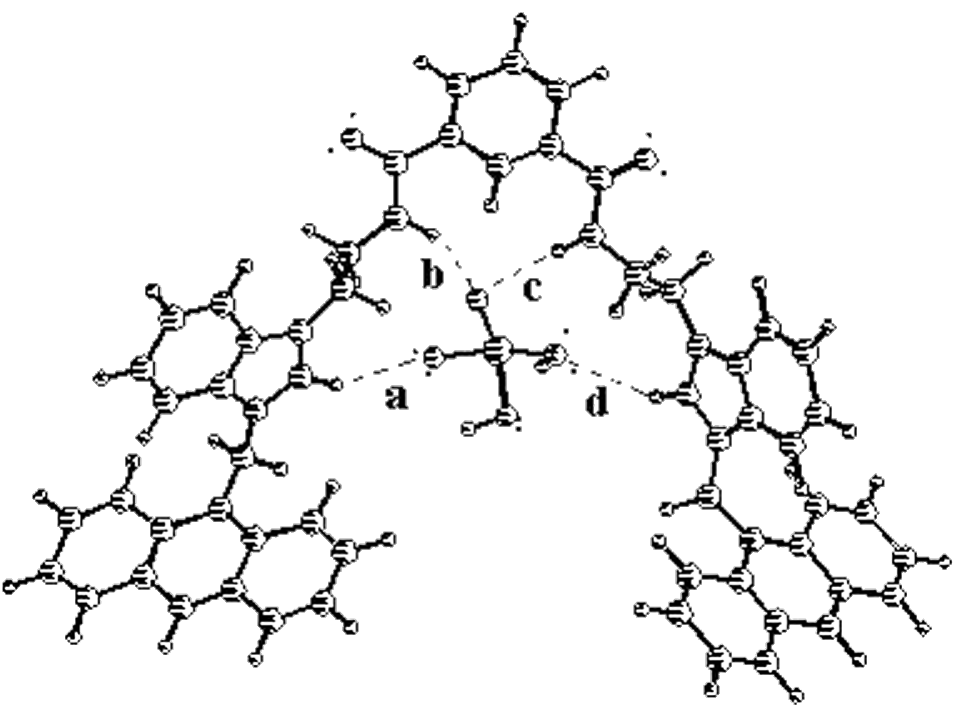
Energy optimized geometry of **1** with H_2_PO_4_^−^ (*E* = 92.14 kcal/mol, *a* = 2.45 Å, *b* = 1.94 Å, *c* = 2.34 Å, *d* = 2.82 Å).

In receptor **1**, two amide NHs and two benzimidazolium protons (C^+^–H) are accessible for the complexation of anions. The residual charge on each benzimidazolium motif, in principle, will stabilize the complex by charge-charge interaction. It is quite rational that the binding group in each arm of flexible receptor **1** could either be involved cooperatively or functions individually for complexation of anions. Thus the selectivity in the binding process is related with the disposition of the binding groups around the isophthaloyl spacer. Figure 2, for example, shows the energy-optimized geometry of the complex of **1** with H_2_PO_4_^−^ in the gas phase [34]. In the complex, benzimidazolium protons (C^+^–H) and amide protons are cooperatively involved in hydrogen bonding with H_2_PO_4_^−^. Anthracene, being a fluorophore in **1**, has the advantage of being considered as a flat hydrophobic fluorophore probe for sensing of anions by the change in intensity due to the photo-induced electron transfer (PET) mechanism.

**Figure 3:**
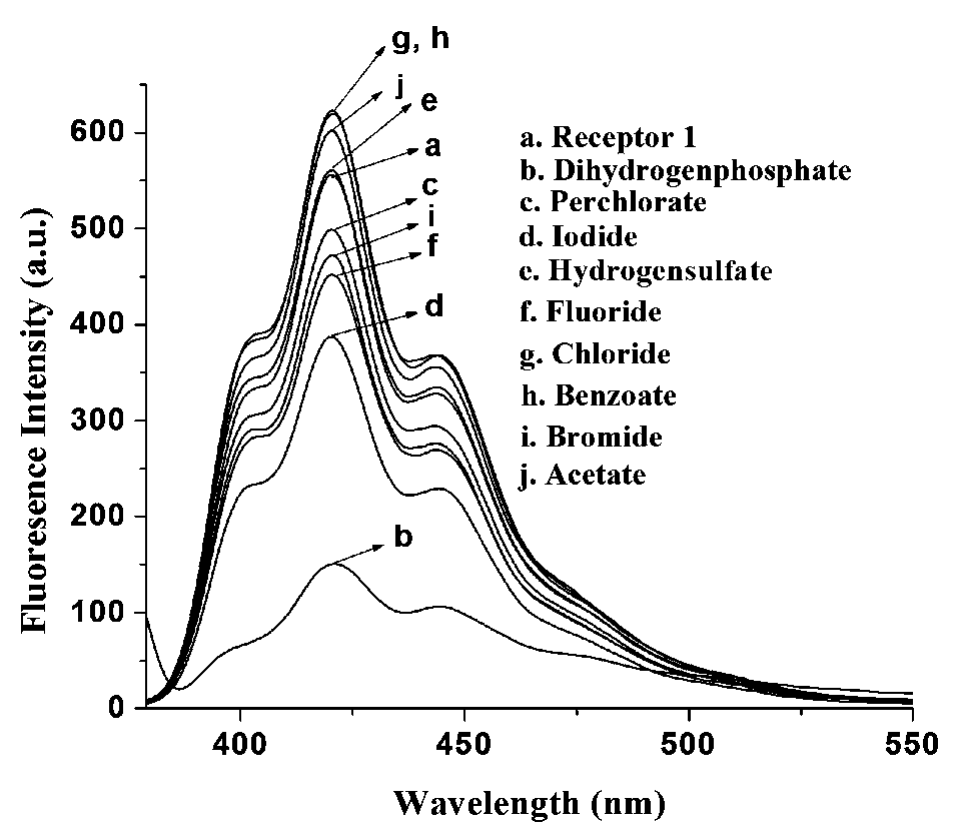
Change in fluorescence emission of **1** (*c* = 5.78 × 10^−5^ M) in the presence of 2 equiv of tetrabutylammonium salts of different guests in CH_3_CN.

**Figure 4:**
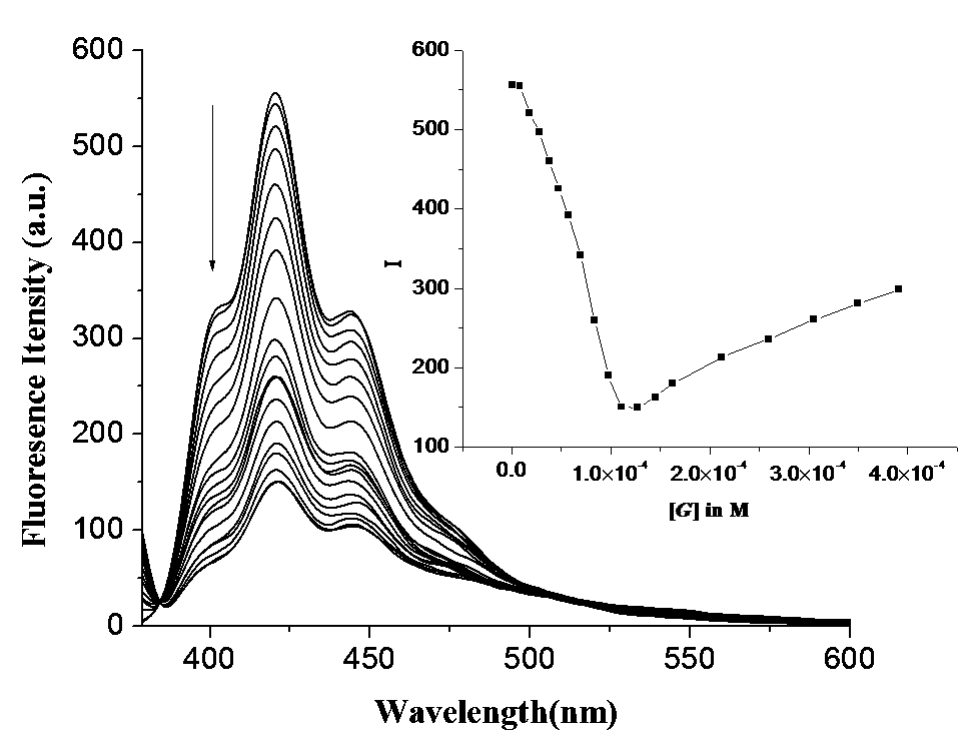
Change in emission spectra of **1** (*c* = 5.78 × 10^−5^ M) in presence of increasing amounts of H_2_PO_4_^−^ in CH_3_CN; Inset: Plot of fluorescence intensity vs concentration of H_2_PO_4_^−^.

As expected, we observed a change in emission of **1** (*c* = 5.78 × 10^−5^ M) in CH_3_CN upon the addition of anions as their tetrabutylammonium salts. Receptor **1** (*c* = 5.78 × 10^−5^ M) in CH_3_CN gave a structured emission band when excited at 369 nm. Upon the addition of 2 equiv of each particular anion to the receptor solution of **1**, a large change in emission of the anthracene group was observed for H_2_PO_4_^−^ (Figure 3): Other anions perturbed the emission of **1** only weakly. Upon the addition of 2 equiv of tetrabutylammonium salts of H_2_PO_4_^−^, F^−^, Br^−^ and I^−^, the emission of **1** was quenched by 72, 18, 14 and 30%, respectively. During titration experiments, no other changes such as excimer or exciplex formation were observed. The large quenching in emission of **1** upon increasing H_2_PO_4_^−^ concentration is illustrated in Figure 4.

**Figure 5:**
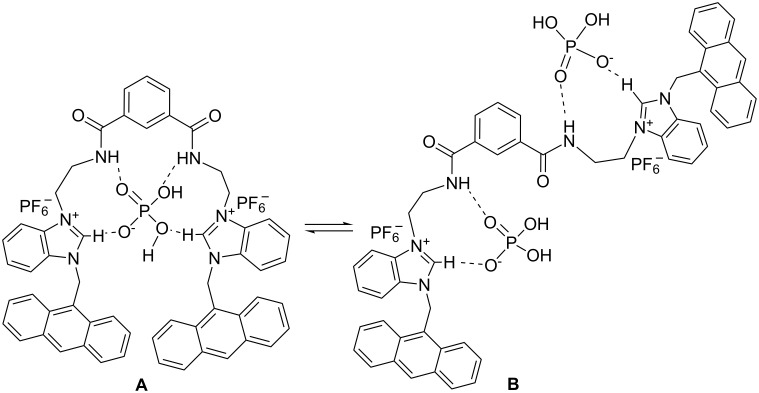
Suggested modes of binding of the H_2_PO_4_^−^ ion into the cleft of **1**.

Interestingly, the emission of **1** is quenched up to the addition of an equivalent amount of H_2_PO_4_^−^ ions. Further addition caused an increase in emission. This is presumably due to a change in conformation upon complexation or decomplexation of anion, although deprotonation of the bound H_2_PO_4_^−^ cannot be ignored [35]. We believe that initially, the binding sites of **1** interact cooperatively to make a 1:1 complex according to the suggested mode **A**, which in turn, changes to mode **B** with 2:1 (guest:host) stoichiometry in the presence of excess H_2_PO_4_^−^ (Figure 5).

**Figure 6:**
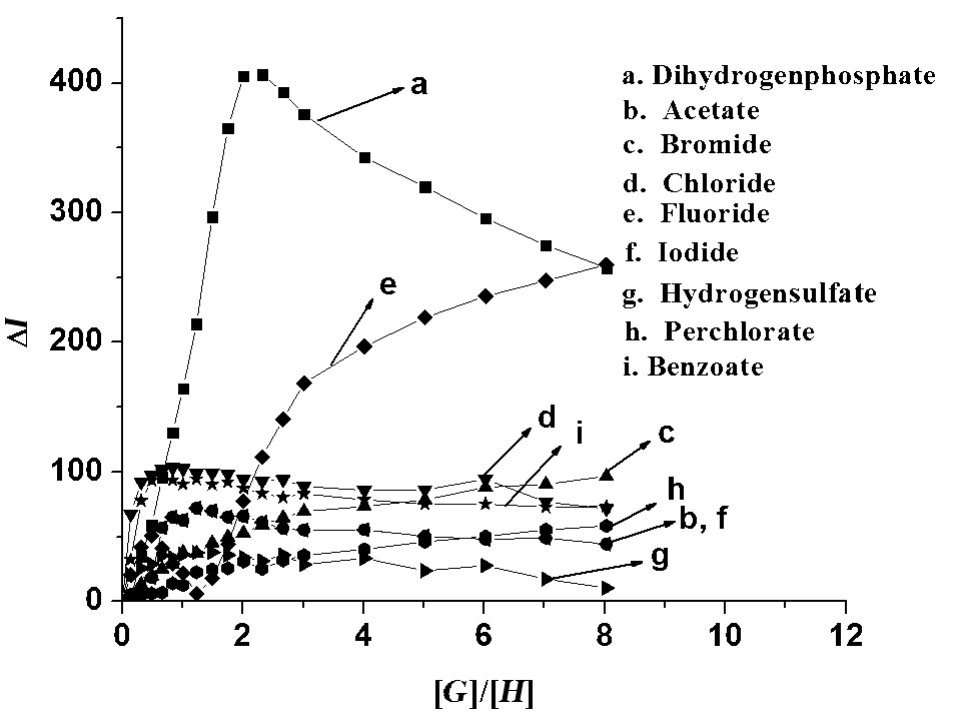
Plot of change in emission of **1** at 420 nm vs the ratio of guest to host concentration in CH_3_CN.

The flexible nature of the binding site contributes to this aspect. For an example of a related system, see [36]. The stoichiometry of the complexes was confirmed by Job plots (see Supporting Information File 1) as well as from the break of the titration curves at [*G*]/[*H*] = 2 (Figure 6).

**Figure 7:**
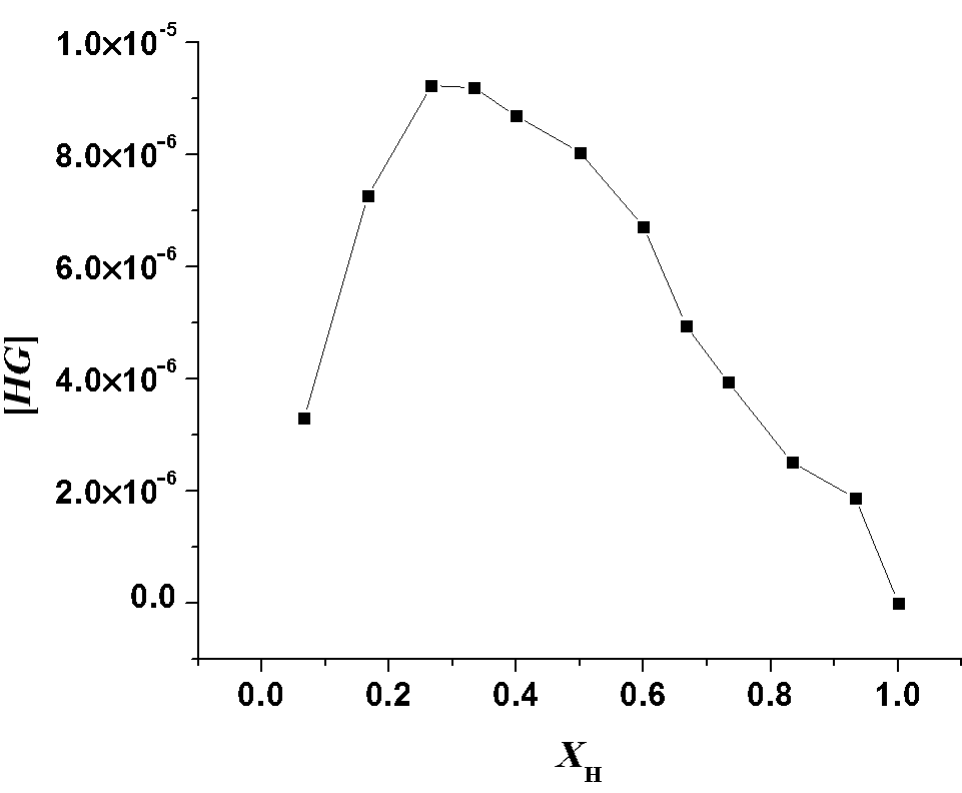
Fluorescence Job plot of **1** with H_2_PO_4_^−^.

Figure 7, for example, represents the Job plot [37] for H_2_PO_4_^−^, which corresponds to a clear-cut case for 2:1 stoichiometry of the complex. In the binding process, cooperative interaction of the two benzimidazolium motifs in **1** are primarily necessary for a large change in emission. This was proved by considering the model compound **2**, where only one binding site is present for interaction. Under similar experimental conditions, the emission of **2** was only slightly changed.

**Figure 8:**
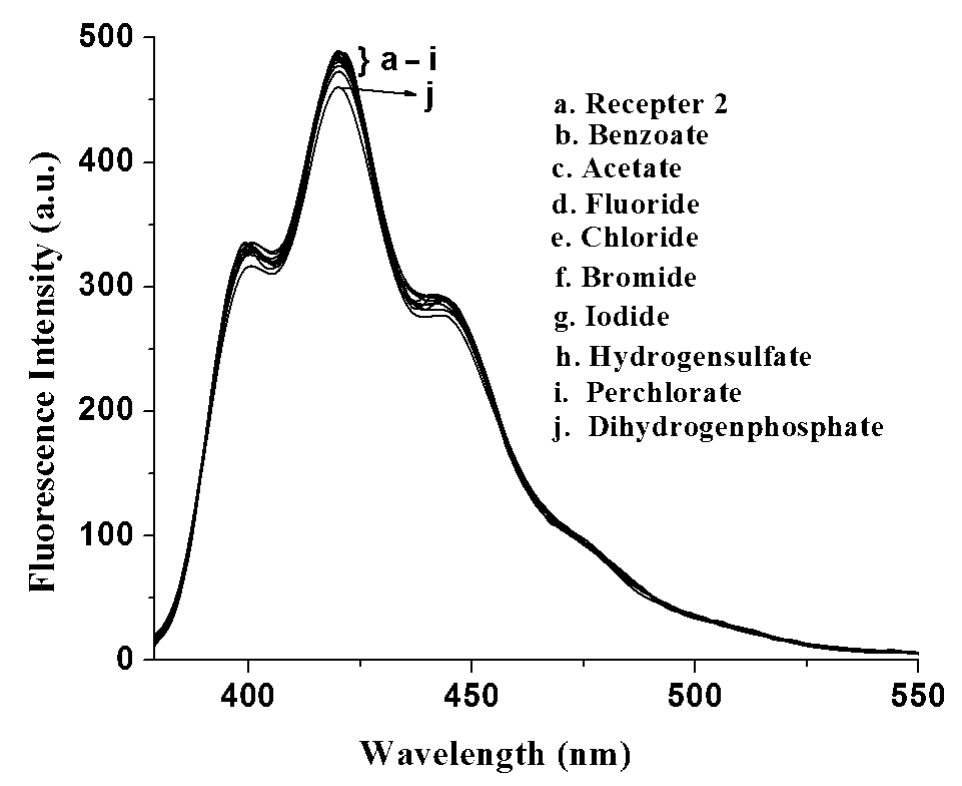
Change in fluorescence emission of **2** (*c* = 3.93 × 10^−5^ M) in the presence of 1 equiv of tetrabutylammonium salts of different guests in CH_3_CN.

Figure 8 displays the change in emission of **2** upon addition of 1 equiv of the same anions in CH_3_CN. In the presence of excess H_2_PO_4_^−^ ions, the change in emission of **2** was less compared to the case of **1** (see Supporting Information File 1).

**Figure 9:**
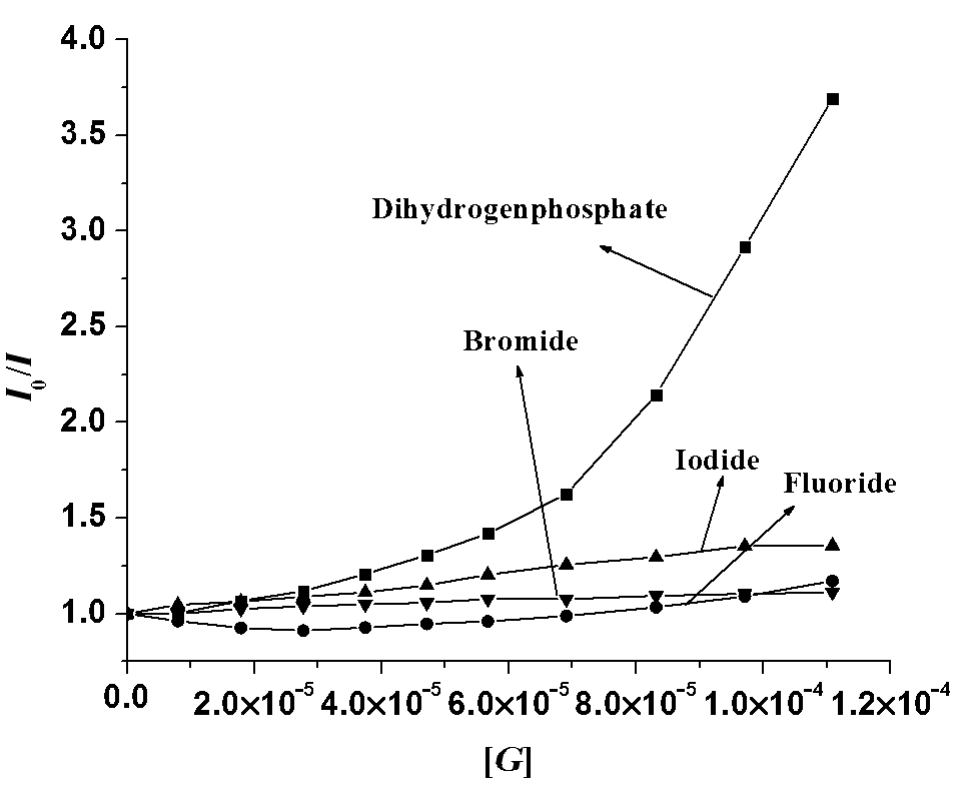
Stern–Volmer plots for **1** (*c* = 5.78 × 10^−5^ M) with H_2_PO_4_^−^, F^−^, Br^−^ and I^−^ at 420 nm (up to the addition of 2 equiv of guest) in CH_3_CN.

The quenching of emission of **1** upon complexation is attributed to the activation of a PET process occurring between the binding site and the excited state of anthracene. The Stern–Volmer plot in Figure 9 illustrates the quenching phenomena with anions such as H_2_PO_4_^−^, F^−-^, Br^−^ and I^−^. The non-linear nature of the curves in Figure 9 indicates that both static (hydrogen bonding effects) and dynamic quenching (bimolecular collision) take place during binding.

**Figure 10:**
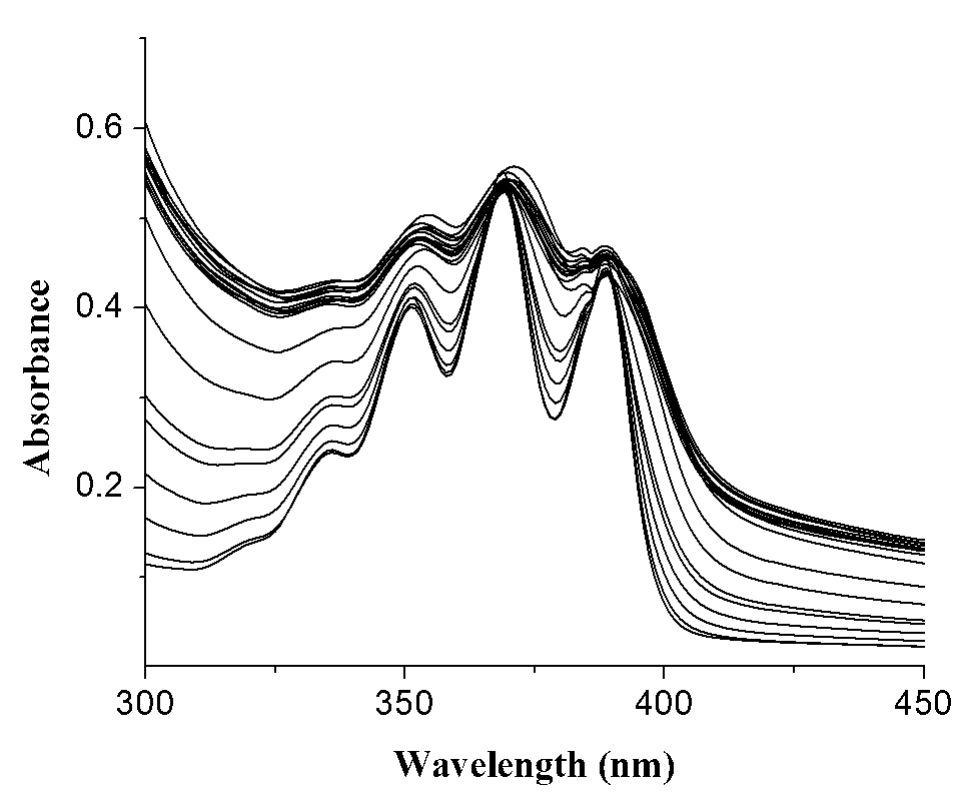
Change in UV–vis spectra of **1** (*c* = 5.78 × 10^−5^ M) in presence of increasing amounts of H_2_PO_4_^−^ in CH_3_CN.

Concurrent UV–vis studies of **1** with the anions exhibited only a small change in absorbance of anthracene and predicted **1** as a PET system [38]. In many cases, the change of absorbance during the titration with the anions was irregular. Figure 10 corroborates the irregular change in absorbance of **1** on titration with H_2_PO_4_^−^ and importantly, in the ground state, all the anions showed 1:1 binding (Supporting Information File 1). However, the selectivity of **1** towards the anions studied was established by determining the binding constant values from fluorescence titration data (Table 1) [39]. As can be seen from Table 1, the receptor **1** shows a marginal selectivity for H_2_PO_4_^−^. The receptor **1** also binds the larger sized iodide ion with 2:1 (guest:host) stoichiometry. By comparison, the change in emission of **1** in the presence of the smaller sized F^−^ is attributed to its greater charge density which causes strong hydrogen bonding followed by deprotonation. Although receptor **1** demonstrates a similar order of binding with F^−^, I^−^ and H_2_PO_4_^−^ in CH_3_CN, the greater fluorometric change of **1** in the presence of H_2_PO_4_^−^ is quite worth mentioning for its fluorometric distinction from other anions in the present study.

**Table 1:** Binding constant values of **1** with the guests by fluorescence method.

Guests^a^	*K*_a_ (M^−1^)^d^ in CH_3_CN	*K*_a_ (M^−1^)^d^ in CHCl_3_ containing 0.1% CH_3_CN

Acetate	—^b^	—^b^
Benzoate	—^b^	—^b^
Dihydrogen phosphate	5.56 × 10^3c^	1.29 × 10^4^
Hydrogen sulfate	—^b^	—^b^
Perchlorate	—^b^	—^b^
Fluoride	4.06 × 10^3c^	—^b^
Chloride	—^b^	—^b^
Bromide	—^b^	5.05 × 10^3^
Iodide	2.41 × 10^3c^	6.90 × 10^4^

^a^Tetrabutylammonium salts were used; ^b^Not determined due to minor change; ^c^Considering *K*_11_; ^d^Error: ≤ ±10%.

**Figure 11:**
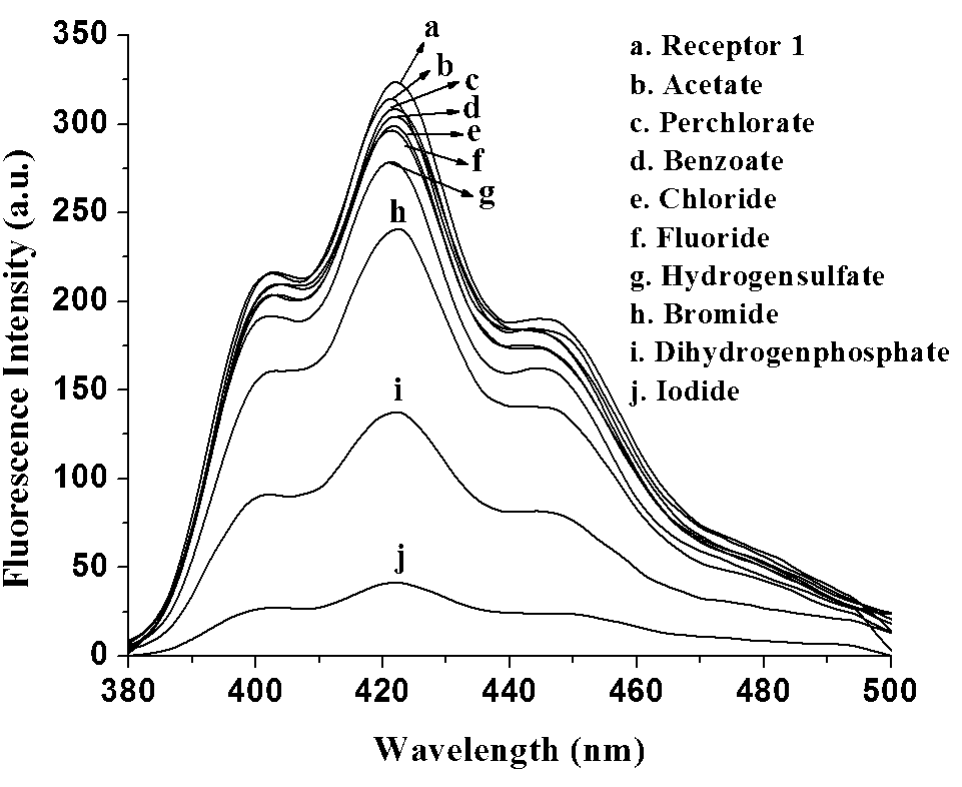
Change in fluorescence emission of **1** (*c* = 5.78 × 10^−5^ M) in the presence of 2 equiv of tetrabutylammonium salts of different guests in CHCl_3_ containing 0.1% CH_3_CN.

For the application of this simple receptor in aqueous system, we carried out the complexation study of **1** in aq CH_3_OH (CH_3_OH:H_2_O = 4:1 v/v) with different phosphate salts. Surprisingly, the change in emission of **1** was found to be negligible (see Supporting Information File 1). This suggested weak or no interactions of **1** in aq CH_3_OH. However, on changing the polarity of the medium both **1** and **2** responded more efficiently and behaved differently. When CH_3_CN was replaced by CHCl_3_ containing 0.1% CH_3_CN, the change in emission of **1** upon complexation of the same anions was very sharp and found to be sharpest for the iodide ion. Figure 11 shows the change in emission of **1** in presence of particular anions. The large quenching of emission in the presence of I^−^ is a characteristic feature of **1** for the fluorometric identification of I^−^ among the other anions in CHCl_3_ containing 0.1% CH_3_CN. Iodide is an important halide that plays an important role in several biological processes such as neurological activity and thyroid function. The iodide content of urine and milk is often required to provide information for nutritional, metabolic, and epidemiological studies of thyroid disorder [40]. In relation to this, very few reports on iodide recognition are known in the literature [41–45].

**Figure 12:**
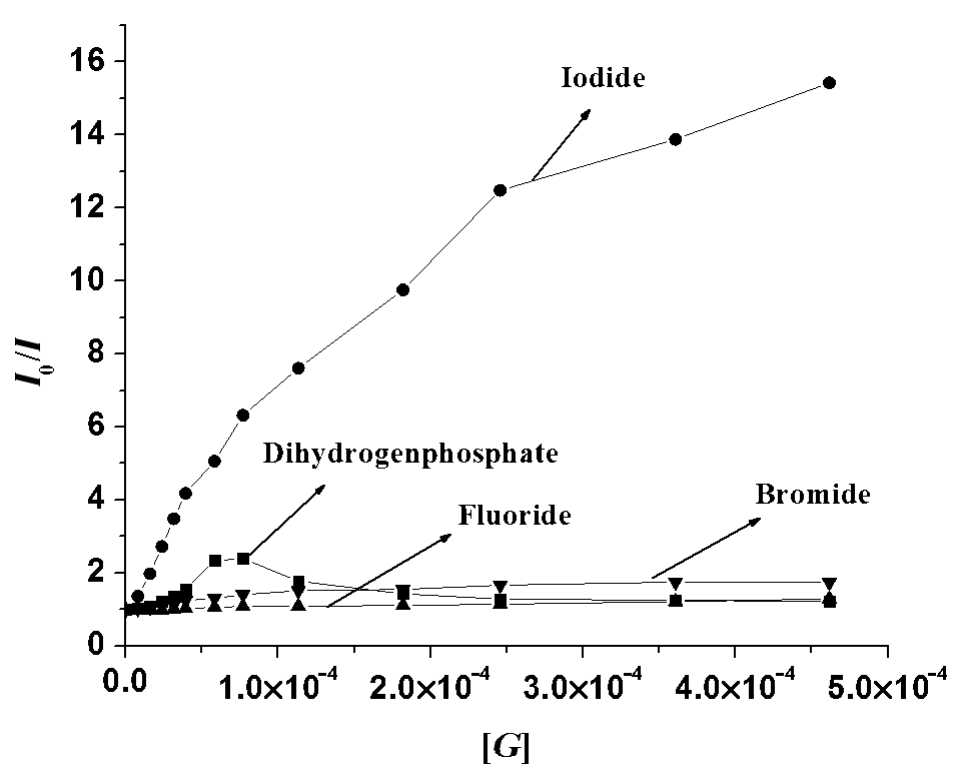
Stern–Volmer plots for **1** (*c* = 5.78 × 10^−5^ M) with H_2_PO_4_^−^, F^−^, Br^−^ and I^−^ at 420 nm (up to the addition of 2 equiv of guest) in CHCl_3_ containing 0.1% CH_3_CN.

Iodide binding induced quenching of emission is attributed to the i) complementarity in size of iodide with the pseudocavity formed by the receptor-binding site and ii) heavy atom effect of iodide, which is also true for Br^−^. But the small quenching of emission in the presence of Br^−^ suggests that I^−^ induced quenching is not only due to the heavy atom effect but also involves some hydrogen bonding effects. Figure 12 is the Stern–Volmer plot of the quenching process. It was also noted that while the monomeric benzimidazolium unit **2** was ineffective in CH_3_CN, it showed measurable changes in emission in CHCl_3_ containing 0.1% CH_3_CN (Supporting Information File 1). These observations thus intimate that solvent polarity is an important aspect for monitoring the sensing behavior of benzimidazolium-based receptors. In our opinion, CH_3_CN in the present case participates in H-bonding with the polar C^+^–H bond of benzimidazolium motif and reduces the possibility of host–guest interactions [46]. This is clearly reflected in the binding constant values in Table 1. Due to the presence of a minimum amount of CH_3_CN in CHCl_3_ the binding constant values for the selected anions are greater in magnitude. In the series I^−^ shows a higher value of *K*_a_. We presume that it is due to the dimension of the open cavity of **1** in CHCl_3_ containing 0.1% CH_3_CN for which I^−^ anion fits sterically with 1:1 stoichiometry. Other complexes in CHCl_3_ containing 0.1% CH_3_CN had also 1:1 stoichiometry.

**Figure 13:**
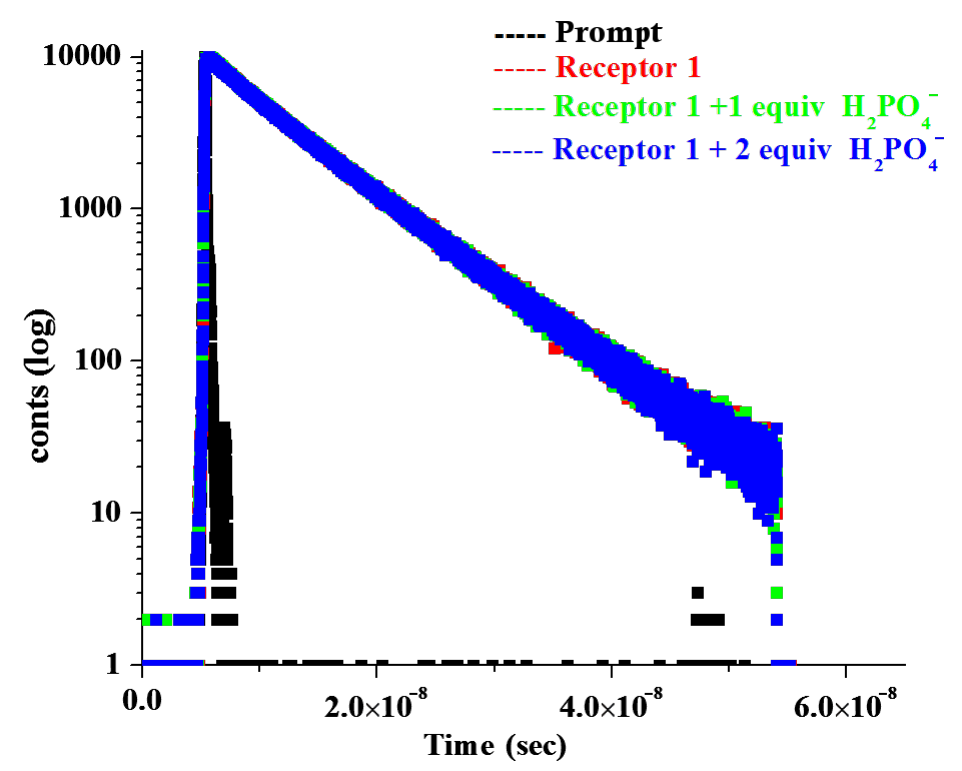
Fluorescence decays (at λ_max_ = 420 nm) of receptor **1** upon the addition of H_2_PO_4_^−^ ion ([*H*] = 5.78 × 10^−5^ M, [*G*] = 4.33 × 10^−3^ M) in CH_3_CN solvent.

**Figure 14:**
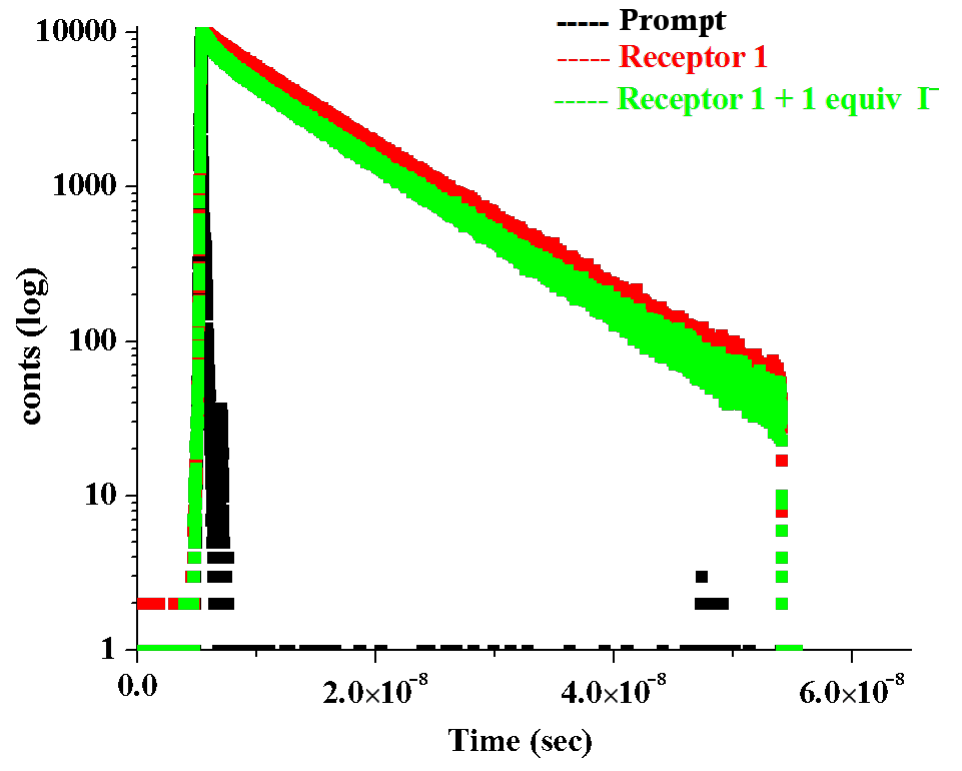
Fluorescence decays (at λ_max_ = 420 nm) of receptor **1** upon the addition of I^−^ ion ([*H*] = 5.78 × 10^−5^ M, [*G*] = 4.55 × 10^−3^ M) in CHCl_3_ containing 0.1% CH_3_CN.

Time resolved fluorescence measurements were additionally carried out to study the dynamics of the flexible receptor **1** (λ_exc_ = 369 nm) in the presence and absence of the anions such as H_2_PO_4_^−^ in CH_3_CN. The emission decay profile of **1** monitored at 420 nm could be fitted bi-exponentially with two constants τ_1_ = 3.42 ns (12.22%), τ_2_ = 7.76 ns (87.78%). The faster decay component (3.42 ns) is due to the anthracene moiety [47] and the relatively stable component with longer lifetime (7.76 ns) is attributed to the benzimidazolium motif of **1**. However, in the presence of 1 equiv of H_2_PO_4_^−^, the lifetime of both the components marginally decreases (Table 2). In the presence of 2 equiv of H_2_PO_4_^−^, the lifetime of the anthracene component increases without showing any marked change in the pre-exponential factor. This was also true for the benzimidazolium component. Figure 13 shows the decay profiles. The change in the lifetimes of the components in **1** in the presence of H_2_PO_4_^−^ ions can be correlated with the change in emission intensity in Figure 4. The fluorescence dynamics of **1** was also carried out for **1** in the presence and absence of I^−^ in CHCl_3_ containing 0.1% CH_3_CN. On changing the solvent combination, the lifetimes of the anthracene and benzimidazolium components of **1** changed (Table 2). Interestingly, while the faster decay component (1.45 ns) due to anthracene moiety contributed a small pre-exponential factor (2%), the relatively stable benzimidazolium component (8.90 ns) had a larger contribution (98%) to the total emission of the molecule. However, upon addition of equivalent amounts of I^−^ the decay profile followed a tri-exponential fitting that indicated three emitting species with lifetimes τ_1_ = 2.15 ns (5.82%), τ_2_ = 8.95 ns (92.86%) and τ_3_ = 0.28 ns (1.32%) (Figure 14). A small increase in the lifetimes of both the anthracene and benzimidazolium moieties in **1** is attributed to the formation of hydrogen bonds with I^−^ in the open cavity of **1**. The faster decay component (0.28 ns) is assumed to be either due to a very short-lived species or an artifact or for tunneling of extra energy to the bulk by a non-radiative pathway [47–48].

**Table 2:** Fluorescence decay times (τ) and pre-exponential factors (c) for **1** in the presence and absence of anions.

	τ_1_ (c)	τ_2_ (c)	τ_3_ (c)	χ^2^

Receptor **1**^a^	3.42 ns (12.22%)	7.76 ns (87.78%)	—	1.13
**1** + Bu_4_N^+^H_2_PO_4_^−^ (1:1)^a^	3.36 ns (11.40%)	7.72 ns (88.60%)	—	1.18
**1** + Bu_4_N^+^H_2_PO_4_^−^ (1:2)^a^	3.49 ns (11.74%)	7.75 ns (88.26%)	—	1.13
Receptor **1**^b^	1.45 ns (2%)	8.90 ns (98%)	—	1.12
**1** + Bu_4_N^+^I^−^ (1:1)^b^	2.15 ns (5.82%)	8.95 ns (92.86%)	0.28 ns (1.32%)	1.10

^a^In CH_3_CN; ^b^In CHCl_3_ containing 0.1% CH_3_CN.

**Figure 15:**
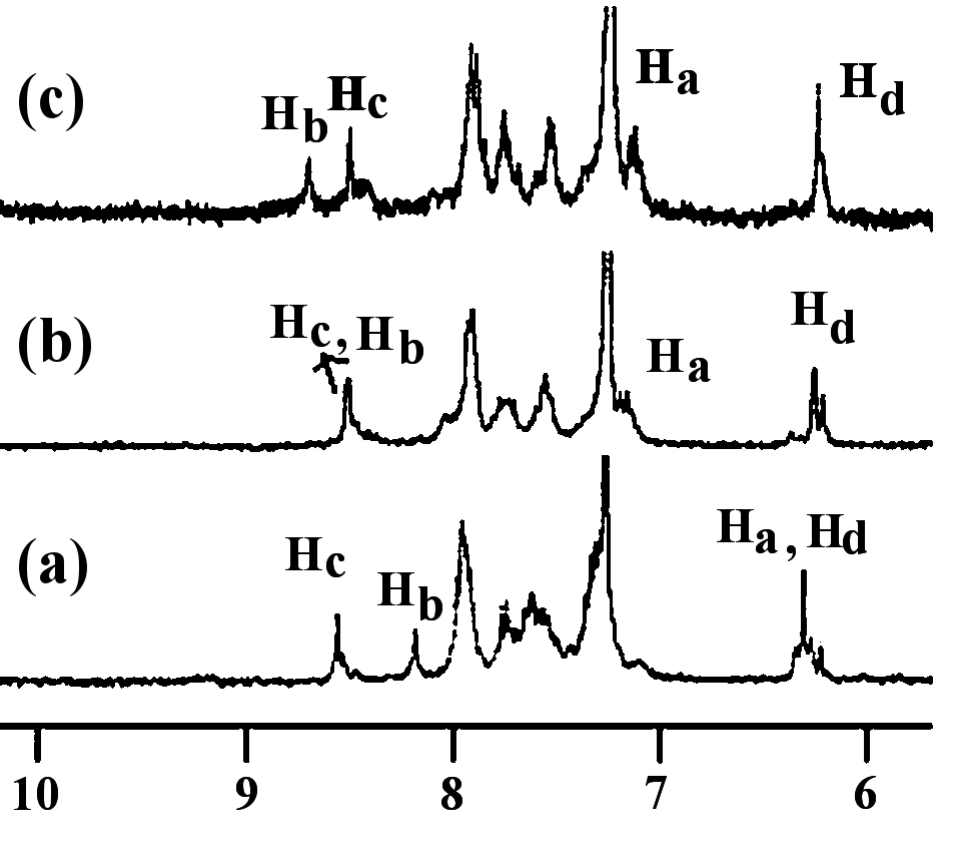
Partial ^1^H NMR (300 MHz, CDCl_3_ containing 4% CD_3_CN) spectra of (a) **1** (*c* = 1.38 × 10^−3^ M), (b) 1:1 and (c) 2:1 (guest:host) complexes with H_2_PO_4_^−^ [see labeled structure **1**].

The expected strong interaction of **1** with H_2_PO_4_^−^ was established additionally by ^1^H NMR. Although the guest is primarily bound by the benzimidazolium motif on account of a charge–charge interaction, there is undoubtedly a degree of cooperation from the amide protons as evidenced by the downfield change in their chemical shift positions in ^1^H NMR. Figure 15 indicates the change in chemical shift of the interacting amides and benzimidazolium protons upon complexation of H_2_PO_4_^−^ ions and shows the broadening of the signals of **1** in the ^1^H NMR spectrum. A precipitate appeared during the course of the study due to insolubility in the NMR concentration range and this was one reason for not determining the binding constant values by the NMR method. However, both the amide and benzimidazolium protons (0.51 ppm) were found to move downfield and thereby supported our binding proposition as indicated in Figure 5. The exact position of amide protons H_a_ was difficult to determine upon complexation. Hydrogen bonding and deprotonation of **1** in the presence of F^−^ was also evidenced from ^1^H NMR (Supporting Information File 1, Figure 11S). In the presence of 1 equiv of F^−^ the benzimidazolium protons in **1** moved downfield by 0.30 ppm which became 0.59 ppm when 2 equiv of F^−^ were added to the receptor solution.

## Conclusion

In conclusion, we have designed a new type of flexible receptor **1**, which uniquely responses the recognition of H_2_PO_4_^−^ and I^−^ by exhibiting large quenching of emission of anthracene group in solvents of different polarities. Two benzimidazolium motifs with an isophthaloyl spacer group in **1,** act cooperatively for successful detection of H_2_PO_4_^−^ in CH_3_CN. In comparison, under the same experimental condition, a single benzimidazolium unit in **2** is inefficient in sensing H_2_PO_4_^−^ ion. By contrast, in the less polar solvent CHCl_3_ containing 0.1% CH_3_CN, the receptor shows a greater fluorescence quenching for I^−^. Such differential quenching of **1** in the presence of H_2_PO_4_^−^ and I^−^ in different solvent combinations is presumably due to the activation of the PET process occurring between the binding site and the excited state of anthracene function at the different rates. We believe at present that the different degrees of solvation of the anions as well as the effective dimension of the binding site which in turn, controls the selectivity in binding process in the excited state are the key factors that regulate the PET to different extents. The binding affinity and selectivity of this simple fluororeceptor are associated with the combined effects of semi-rigid structures of receptor, charge-charge interactions, and the involvement of both N–H---O and C–H---O hydrogen bonds. Further work is underway in our laboratory.

## Experimental

### General methods

Solvents were distilled prior to use, and dried according to the literature procedure when required. Chromatographic separations were performed on silica gel (60–120 mesh). All melting points were determined in open capillaries and are uncorrected. ^1^H NMR spectra were recorded on Bruker 400 and 300 MHz spectrometers. ^13^C NMR spectra were recorded on a Bruker 400 MHz spectrometer. FT IR spectra were recorded on a Perkin-Elmer L120-00A spectrometer as KBr discs. UV–vis spectra were recorded on Perkin-Elmer Lambda-25 and fluorescence spectra on a Perkin-Elmer LS 55 spectrofluorometer, respectively.

### Synthesis of 3 [49]

To a solution of benzimidazole (0.300 g, 2.54 mmol) in dry THF (15 mL), NaH (0.14 g) was added and the mixture refluxed for 1 h under a nitrogen atmosphere. The reaction mixture was then cooled to room temperature, 9-chloromethylanthracene (0.700 g, 3.09 mmol) in THF (15 mL) added and then heated under reflux for 10 h. The THF was removed, water added and the mixture extracted with CHCl_3_ (3 × 30 mL). The organic layer was dried over anhydrous Na_2_SO_4_ and concentrated on a rotary evaporator. Purification of the crude product by silica gel column chromatography with 20% ethyl acetate in petroleum ether as eluent gave compound **3** (0.500 g, yield 64%). ^1^H NMR in CDCl_3_ (400 MHz) δ 8.61 (s, 2H), 8.10 (d, 4H, *J* = 8 Hz), 7.82 (d, 1H, *J* = 8 Hz), 7.71 (d, 1H, *J* = 8 Hz), 7.51 (m, 4H), 7.42 (t, 1H, *J* = 8 Hz), 7.35(t, 1H, *J* = 8 Hz), 6.19 (s, 2H); ^13^C NMR (CDCl_3_, 100 MHz) δ 144.0, 142.2, 134.2, 131.4, 131.0, 129.7, 129.5, 127.5, 125.4, 123.6, 123.1, 123.0, 122.5, 120.6, 109.5, 41.3; *m/z* (ES^+^): 308.9 [M]^+^.

### Synthesis of 4

A mixture of 2-chloroethylamine hydrochloride (704 mg, 6 mmol) in dry CH_2_Cl_2_ (30 mL) containing triethylamine (1.35 mL) was stirred for 1 h to give the free amine. To this solution, isophthaloyl dichloride (600 mg, 2.9 mmol) was added and the reaction mixture stirred at room temperature for 8 h. The solvent was evaporated and water added. The aqueous layer was extracted with CHCl_3_ (3 × 50 mL), the combined organic phases was washed with water, dried over anhydrous Na_2_SO_4_ and concentrated in vacuo. The resulting crude solid was purified by column chromatography with 50% ethyl acetate in petroleum ether as eluent to give pure **4** (0.67 g, yield 78%), mp 145 °C, ^1^H NMR (CDCl_3,_ 400 MHz): δ 8.25 (s, 1H), 7.94 (dd, 2H, *J*_1_ = 8 Hz, *J*_2_ = 4 Hz), 7.53 (t, 1H, *J* = 8 Hz), 6.84 (br t, 2H, -NH-), 3.84–3.79 (m, 4H), 3.75–3.71 (m, 4H); ^13^C NMR (CDCl_3_ containing few drops DMSO-*d*_6_, 100 MHz) δ 166.0, 134.21, 129.8, 128.3, 126.2, 42.8, 41.3; FTIR: ν cm^−1^ (KBr): 3288, 3066, 2915, 2863, 1639, 1539; *m*/*z* (LCMS): 289 (M)^+^.

### Synthesis of 1

To a stirred solution of **4** (0.1 g, 3.8 mmol) in dry CH_3_CN (20 mL), compound **3** (0.236 g, 7.6 mmol) was added and the reaction mixture heated under reflux for 3 days. During the heating the dichloride salt **5** precipitated and was removed by filtration. Repeated recrystallization of the salt from CH_3_CN gave almost pure **5** (0.184 g, 59.7% yield). Compound **5** (0.184 g) was then dissolved in hot CH_3_OH (20 mL) followed by addition of NH_4_PF_6_. After stirring the reaction mixture for 30 min, the CH_3_OH was evaporated to reduce the volume. The precipitated salt was filtered, washed with cold water and dried. Repeated recrystallization of **1** from CH_3_CN afforded the pure **1** (0.21 g, 90% yield), mp 210 °C, ^1^H NMR (DMSO-*d*_6,_ 400 MHz): δ 8.94 (s, 2H), 8.83 (s, 2H), 8.50–8.10 (m, 14H), 7.78–7.72 (m, 4H), 7.59–7.49 (m, 12H), 6.63 (s, 4H), 4.46 (br s, 4H), 3.59 (br s, 4H) [due to solubility problem signals were broad in nature]; ^13^C NMR (DMSO-*d*_6_, 100 MHz): 141.8, 134.3, 132.2, 132.0, 131.5, 131.47, 131.43, 131.1, 130.8, 129.7, 128.2, 128.1, 127.3, 127.1, 126.0, 125.9, 123.7, 122.0, 114.6, 114.1, 47.0, 43.7, 38.7; FTIR: ν cm^−1^ (KBr): 3433, 3146, 2954, 1721, 1652, 1565, 1531; m/z (ES^+^): 1123.7 (M − 1)^+^, 979.5 [(M − PF_6_^−^) ]^+^.

### Synthesis of 2

A mixture of 2-chloroethylamine hydrochloride (460 mg, 3.9 mmol) in dry CH_2_Cl_2_ (20 mL) containing triethylamine (1 mL) was stirred at room temperature for 1 h to give the free amine. Acetyl chloride (0.582 mL, 5.9 mmol) was added to the reaction mixture which was then stirred at room temperature for 8 h. After completion of the reaction, the solvent was evaporated and the residue extracted with CHCl_3_ (3 × 20 mL). The organic extracts were washed with NaHCO_3_ solution (3 × 15 mL) and dried over anhydrous Na_2_SO_4_. The solvent was removed under vacuum and the residue purified by silica gel column chromatography with 80% ethyl acetate in petroleum ether as eluent to afford the compound **6** (0.309 g, 64% yield). The chloro-amide **6** (150 mg, 1.2 mmol) was heated under reflux in dry CH_3_CN (20 mL) with the anthracene-coupled benzimidazole **3** (570 mg, 1.85 mmol) for 4 days. The precipitated chloride salt was filtered, washed with water and dried (0.51 g, 64.4%). The compound **6** in MeOH was subsequently treated with aqueous NH_4_PF_6_ solution to carry out the anion exchange reaction. The solution was heated with stirring for 20 min until a precipitate appeared. Filtration of the precipitate followed by thorough washing with ether afforded compound **2** in 95% yield (0.61 g), mp 203 °C (decomposition); ^1^H NMR (DMSO-*d*_6,_ 400 MHz): δ 8.93 (s, 1H), 8.82 (s, 1H), 8.41 (d, 1H, *J* = 8 Hz), 8.33 (d, 2H, *J* = 8 Hz), 8.27 (d, 2H, *J* = 8 Hz), 8.08 (d, 1H, *J* = 8 Hz) 7.84–7.78 (m, 2H), 7.63 (br m, 5H), 6.67 (s, 2H), 4.32 (br s, 2H), 3.50 (br s, 2H), 2.32 (s, 3H); ^13^C NMR (DMSO-*d*_6_, 100 MHz): 169.4, 141.2, 131.8, 131.4, 131.1, 131.0, 130.4, 129.3, 127.8, 126.8, 126.6, 125.6, 123.3, 121.5, 114.2, 113.5, 57.4, 46.4, 43.2, 37.2; FTIR: ν cm^−1^ (KBr): 3436, 1718, 1654, 1563, 1453. *m*/*z* (LCMS): 394.2 [(M − PF_6_^−^) ]^+^.

**General procedure of fluorescence and UV–vis titrations**: Stock solutions of the receptors were prepared in different solvents such as CH_3_CN and CHCl_3_ containing 0.1% CH_3_CN and 2.5 ml of the individual receptor solution was placed in the cuvette. Stock solutions of anions were prepared in the same solvents, and were added individually in different amounts to the receptor solution. For fluorescence, the receptor solutions prepared in dry CH_3_CN were irradiated at the excitation wavelength 369 nm maintaining the excitation and emission slits 8 and 8, respectively and the filter was 1% attenuated. Upon the addition of anions, the change in emission and absorbance of the receptors were noted. Similarly, for emission, the receptors dissolved in CHCl_3_ containing 0.1% CH_3_CN were irradiated at the excitation wavelength 369 nm maintaining the excitation and emission slits 10 and 5, respectively.

**Method for Job plots**: The stoichiometry was determined by the continuous variation method (Job Plot) [35]. In this method, solutions of host and guests of equal concentrations were prepared in dry CH_3_CN and CHCl_3_ containing 0.1% CH_3_CN. Then the host and guest solutions were mixed in different proportions maintaining a total volume of 3 mL of the mixture. The related compositions for host:guest (*v/v*) were 3:0, 2.8:0.2, 2.5:0.5, 2.2:0.8, 2:1, 1.8:1.2, 1.5:1.5, 1:2, 0.8:2.2, 0.5:2.5, 0.2:2.8. All the prepared solutions were kept for 1 h at room temperature with occasional shaking. Then the emission and absorbance of the solutions of different compositions was recorded. The concentration of the complex, i.e., [*HG*] was calculated using the equation [*HG*] = Δ*I*/*I*_0_ × [*H*] or [*HG*] = Δ*A*/*A*_0_ × [*H*] where Δ*I*/*I*_0_ and Δ*A*/*A*_0_ indicate the relative emission and absorbance intensities. [*H*] corresponds to the concentration of pure host. The mole fraction of the host (*X*_H_) was plotted against the concentration of the complex [*HG*]. In the plot, the mole fraction of the host at which the concentration of the host–guest complex concentration [*HG*] is maximum, gives the stoichiometry of the complex.

## Supporting Information

File 1 Supplementary Data.
