# Supplementary material for: Fluorometric recognition of both dihydrogen phosphate and iodide by a new flexible anthracene linked benzimidazolium-based receptor
Source: Beilstein J Org Chem. 2011 Feb 25;7:254–64. doi: 10.3762/bjoc.7.34 (PMC3063073; doi:10.3762/bjoc.7.34)
Supplement: File 1 — Supplementary Data. [file Beilstein_J_Org_Chem-07-254-s001.pdf]

Supporting Information

for

Fluorometric recognition of both dihydrogen phosphate and iodide by a new flexible anthracene linked benzimidazolium-based receptor

Kumaresh Ghosh[*§] and Debasis Kar

Department of Chemistry, University of Kalyani, Kalyani-741235, India.

Email: Kumaresh Ghosh - ghosh_k2003@yahoo.co.in

[§]Fax: +913325828282; Tel: +913325828750

*Corresponding author

Supplementary Data

1. Absorption spectra

(a) (b)

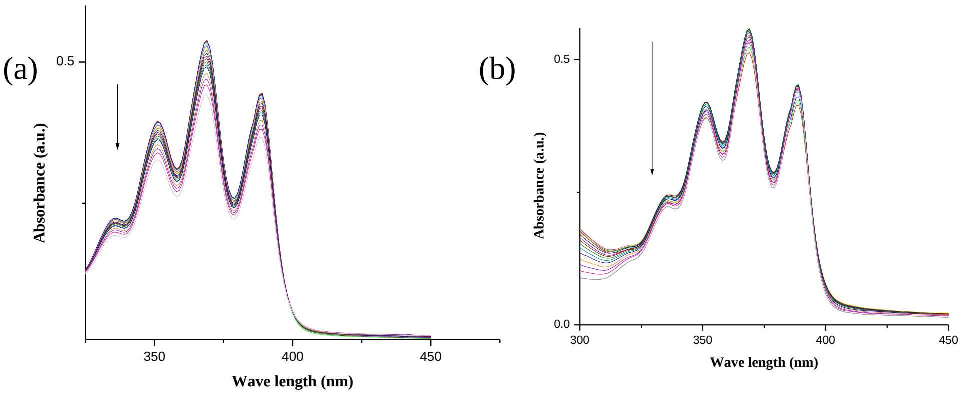

Figure 1S: Change in absorption spectra of **1** ($c = 5.78 \times 10^{-5}$ M) on titration with (a) F^- and (b) I^-.

2. Fluorescence spectra

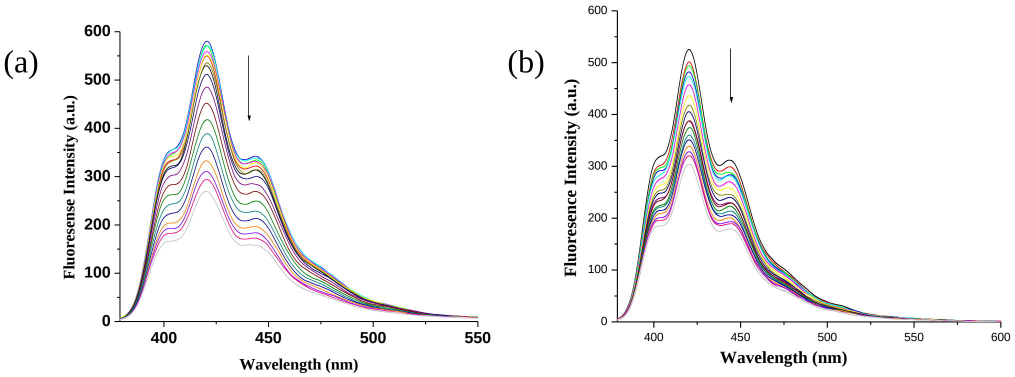

Figure 2S: Change in emission spectra of **1** ($c = 5.78 \times 10^{-5}$ M) on titration with (a) F^- and (b) I^- in CH_3CN.

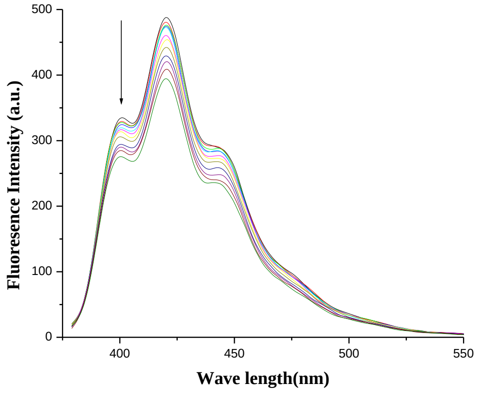

Figure 3S: Change in emission spectra of **2** ($c = 5.78 \times 10^{-5}$ M) in presence of increasing amounts of tetrabutylammonium dihydrogen phosphate in CH_3CN.

3. Fluorescence Job plots in CH_3CN.

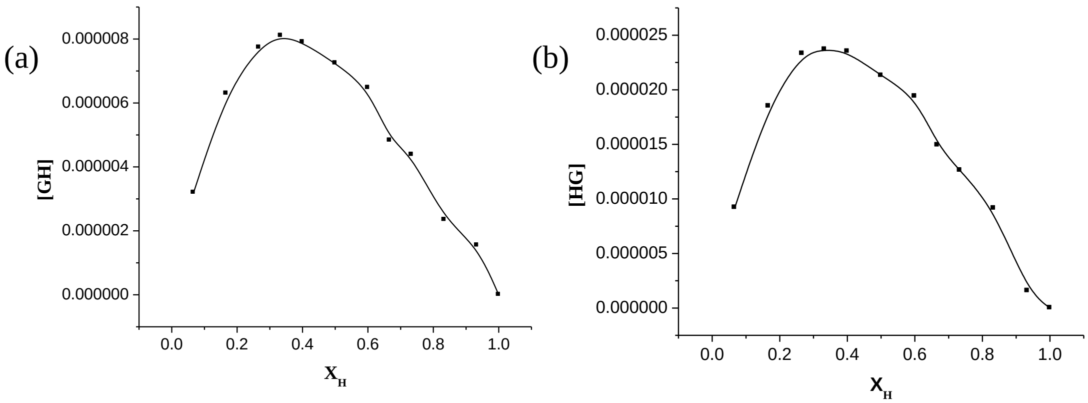

Figure 4S: Fluorescence Job plots for **1** with (a) fluoride and (b) iodide.

4. UV–vis titration curves for 1 with the anions in CH_3CN.

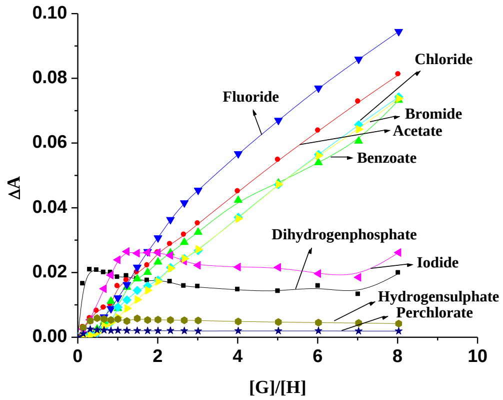

Figure 5S: Plot of change in absorbance of **1** at 369 nm vs the ratio of guest to host concentration in CH_3CN.

5. Binding constant curves

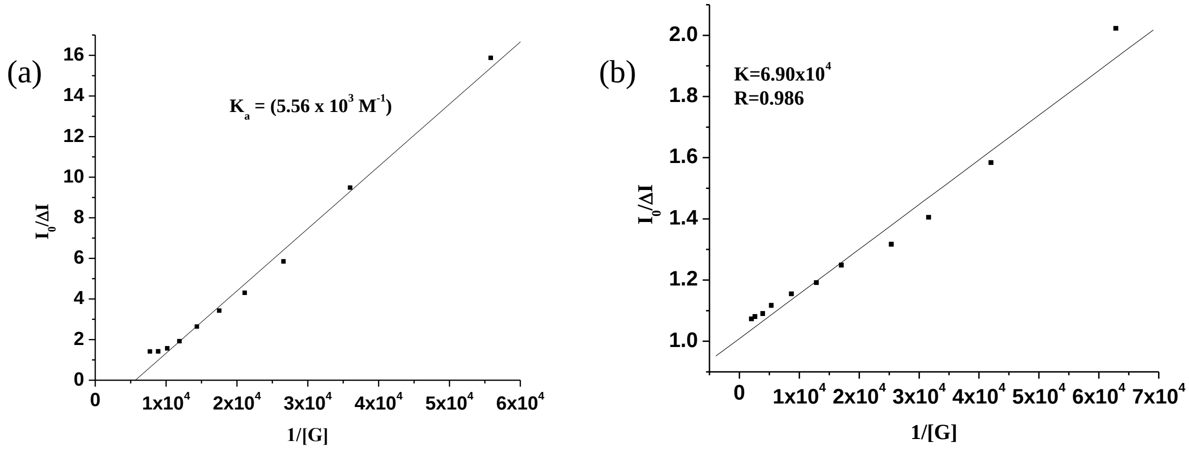

Figure 6S: Binding constant curves for **1** with (a) $H_2PO_4^-$ in CH_3CN and (b) iodide in $CHCl_3$ containing 0.1% CH_3CN.

6. Change in emission of 1 in aq CH_3OH ($CH_3OH:H_2O$ = 4:1 v/v).

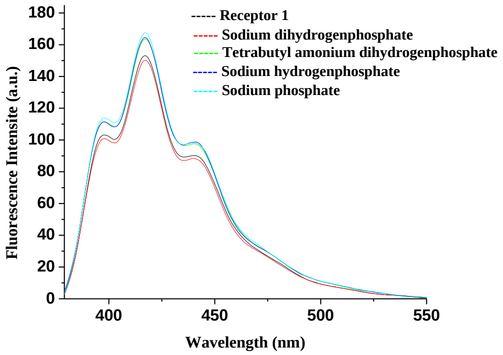

Figure 7S: Change in fluorescence emission of **1** ($c = 2.46 \times 10^{-5}$ M) in the presence of 1 equiv of the salts of different guests in aq CH_3OH.

7. (a) Fluorescence titration curves for 1 with the anions in $CHCl_3$ containing 0.1% CH_3CN.

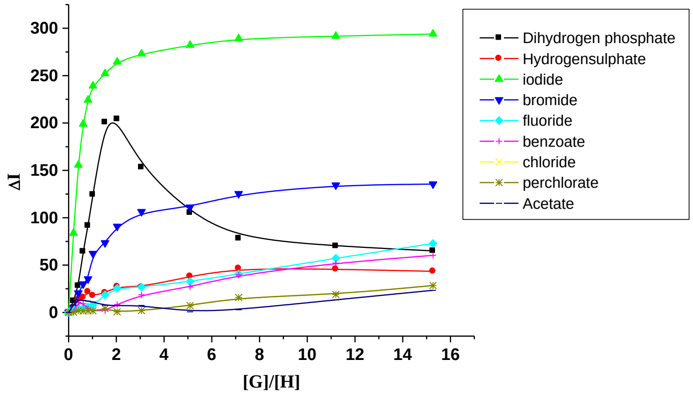

Figure 8S: Plot of change in emission of **1** at 420 nm vs the ratio of guest to host concentration.

(b) Fluorescence titration curves for 2 with the anions in $CHCl_3$ containing 0.1% CH_3CN.

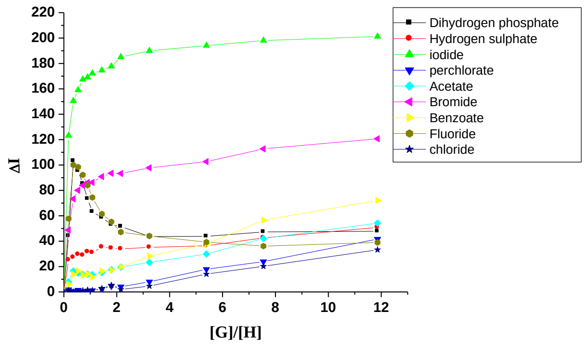

Figure 9S: Plot of change in emission of **2** vs the ratio of guest to host concentration.

8. Fluorescence Job plots anions in $CHCl_3$ containing 0.1% CH_3CN.

(a)

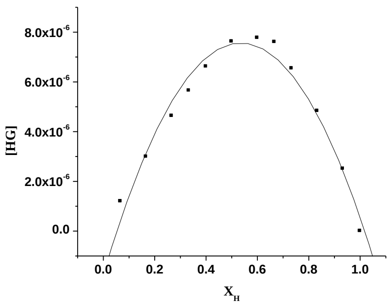

(b)

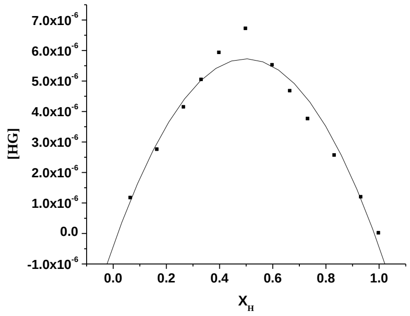

Figure 10S: Fluorescence Job plots for **1** with (a) iodide and (b) dihydrogen phosphate.

9. Change in ^{1}H NMR of 1 in the presence of F^-.

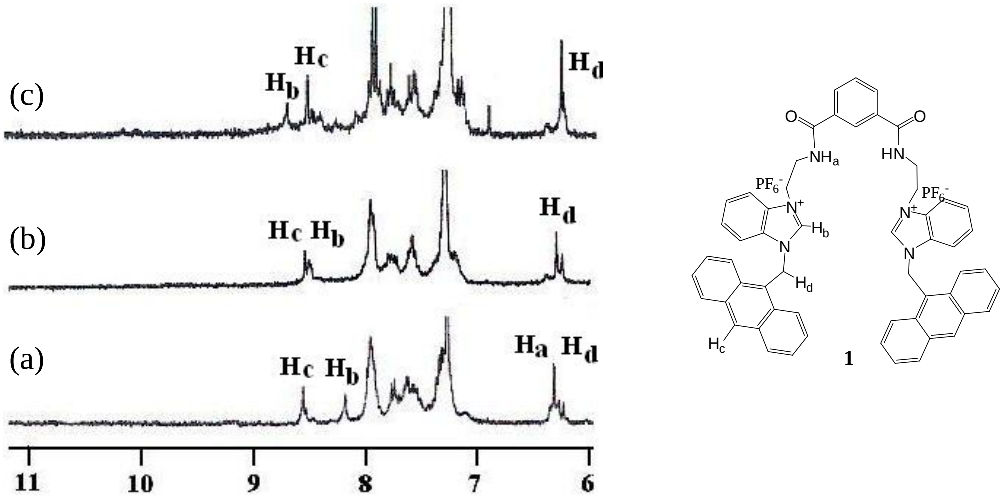

Figure 11S: Partial ^{1}H NMR (300 MHz, $CDCl_3$ containing 4% CD_3CN) spectra of (a) **1** ($c = 1.36 \times 10^{-3}$ M), (b) 1:1 and (c) 2:1 (guest:host) complexes with F^-; Note: After 1 equiv addition of F^- the amide protons H_a of **1** were difficult to identify correctly.
